# Proteomic Analysis of the Effect of Acupuncture on the Suppression of Kainic Acid-Induced Neuronal Destruction in Mouse Hippocampus

**DOI:** 10.1155/2013/436315

**Published:** 2013-07-23

**Authors:** Chang-Hwan Bae, Dong-Soo Kim, Ye Lee Jun, Sunoh Kwon, Hi-Joon Park, Dae-Hyun Hahm, Hyejung Lee, Seung-Tae Kim

**Affiliations:** ^1^Division of Meridian and Structural Medicine, School of Korean Medicine, Pusan National University, Yangsan 626-870, Republic of Korea; ^2^Acupuncture & Meridian Science Research Center, Kyung Hee University, Seoul 130-701, Republic of Korea; ^3^Department of Psychiatry and Behavioral Sciences, Northwestern University Feinberg School of Medicine, Chicago, IL 60611, USA

## Abstract

Kainic acid (KA) is a neurotoxin that induces epileptic seizures and excitotoxicity in the hippocampus. Acupuncture is frequently used as an alternative therapy for epilepsy, and it has been known to protect hippocampal neurons against KA toxicity. Using proteomic analysis, we investigated protein expression changes in the hippocampus following acupuncture stimulation at HT8. Eight-week-old male C57BL/6 mice (20–25 g) received acupuncture treatment at HT8 acupoint bilaterally once a day for 3 days and were then administered KA (30 mg/kg) intraperitoneally. Twenty-four hours after KA injection, neuronal survival and astrocyte activation in the hippocampus were measured, and protein expression in the hippocampus was identified by 2-dimensional electrophoresis. Acupuncture stimulation at HT8 suppressed KA-induced neuronal death and astrocyte activation in the hippocampus. We identified the changes in the expression of 11 proteins by KA or acupuncture stimulation at HT8 and found that acupuncture stimulation at HT8 normalized the expression of dihydropyrimidinase-related protein 2 and upregulated the expression of transcriptional activator protein pur-alpha, serine/threonine-protein phosphatase 5, and T-complex protein 1 subunit alpha, which are related to the survival of neurons. These results suggest that acupuncture stimulation at HT8 changes protein expression profiles in the hippocampus in favor of neuronal survival in KA-treated mice.

## 1. Introduction

Kainic acid (KA) is a potent agonist to the *α*-amino-3-hydroxy-5-methyl-4-isoxazolepropionic acid (AMPA)/kainate receptor. The administration of KA commonly induces epileptic seizures and excitotoxic cell death in the hippocampus, similar to that observed in temporal lobe epilepsy patients [1, 2]. Therefore it has been widely used for seizure animal studies as a trigger to induce temporal lobe epilepsy in animal models [3].

Acupuncture has been used to alleviate various kinds of neurological disorders, including epilepsy and seizure [4], but the mechanism of acupuncture for epilepsy treatment remains elusive. Among acupoints, HT8 has been used as a therapeutic acupoint for epilepsy. In KA-induced epileptic seizure animal studies, acupuncture stimulation at HT8 reduces the severity of epileptic seizures, regulates GABA-mediated signaling by increasing glutamate decarboxylase [5], and suppresses KA-induced hippocampal cell death by suppressing microglia activation and cytokines [6]. These results indicate that acupuncture stimulation at HT8 protects hippocampal cells from KA toxicity, but it is still unknown which proteins are related to the action of the acupuncture stimulation.

Two-dimensional electrophoresis (2-DE) is a method that separates proteins based on electrical charge and molecular weight from complex protein mixtures extracted from biological samples such as cells and tissues, and this method can detect changes in protein expression levels and isoforms. Using this technique, we investigated the acupuncture-associated proteomic profile changes in the hippocampus in KA-induced mouse model of epilepsy.

## 2. Methods

### 2.1. Animals and Grouping

Male C57BL/6 mice (8 weeks old, weighing 20–23 g; Orient Bio, Inc., Republic of Korea) were housed at room temperature (22  ±  3°C) under a standard 12 h light/dark cycle (lights on at 07:00 h) with unlimited access to food and water. The animals were handled in accordance with the current guidelines established in the NIH Guide for the Care and Use of Laboratory Animals (NIH Publication no. 85-23, 1985), and all efforts were made to minimize animal suffering and reduce the number of animals used. The mice were randomly assigned to four groups: the saline group (*n* = 9) was injected with normal saline and did not receive acupuncture stimulation; the KA group (*n* = 9) was injected with KA (Sigma, St. Louis, MO) and did not receive acupuncture stimulation; the KA + HT8 group (*n* = 9) was injected with KA and received acupuncture stimulation bilaterally at acupoint HT8; and the KA + ST36 group (*n* = 6) was injected with KA and received acupuncture stimulation bilaterally at acupoint ST36 as the control group for acupuncture stimulation.

### 2.2. Acupuncture Stimulation

Between 10:00 and 10:30 a.m., the mice in the KA + HT8 group were lightly immobilized by an assistant who grasped the loose skin behind the ears with thumb and forefinger, and acupuncture needles (0.18 × 8 mm; Dongbang Acupuncture, Inc., Republic of Korea) were inserted at the bilateral HT8 acupoints. HT8 is located on the palmar surface of the forelimbs, between the fourth and fifth metacarpal bones [7]. The needle was inserted to a depth of 1 mm and was turned at a rate of two spins and counter-spins per second for 30 s, with removal immediately afterward. The entire stimulation lasted 60 s. For the KA + ST36 group, the same procedure was applied to acupoint ST36. ST36 is located at the proximal one-fifth point on the line from the depression lateral to the patella ligament to the anterior side of the ankle crease [7]. The positions of the acupoints in mice correspond anatomically to their locations in humans. The stimulation was repeated three times: once a day for 3 days. The animals in the saline and KA groups were immobilized in a similar fashion for the 60 s. 

### 2.3. Kainic Acid Injection

Thirty minutes after the last acupuncture stimulation, KA (30 mg/kg of free base; Sigma) was injected intraperitoneally with a BD Ultra-fine II insulin syringe (Becton, Dickson, and Company, Franklin Lakes, NJ, USA) into the mice in the KA, KA + HT8, and KA + ST36 groups. The mice in the saline group were injected with normal saline instead of KA.

### 2.4. Immunohistochemistry

Twenty-four hours after the KA injection, the mice (*n* = 6 from each group) were perfused with 4% paraformaldehyde dissolved in 0.1 M phosphate buffer (PB). The brains were removed from the craniums, postfixed for a day, washed in 0.1 M PB, and immersed in 30% sucrose solution for storage at 4°C prior to sectioning. Frozen sections (40 *μ*m) were cut using a Leica CM3050S cryostat (Leica microsystems, Wetzlar, Germany). 

To identify degenerating neurons in the hippocampus, cresyl violet staining was performed. The sections were mounted on silane-coated slides, air-dried, and incubated for 1 min in a 1% solution of cresyl violet. Next, the sections were washed thoroughly in cold tap water, rinsed briefly in 1% acetic acid solution for 10 s, dehydrated by immersion in ascending grades of alcohol, cleared with xylene, and coverslipped using mounting medium.

To detect the activation of astrocytes in the hippocampus, the sections were incubated with glial-GFAP (Cell Signaling Technology, Beverly, MA, USA) primary antibodies diluted 1 : 1000 for 24 h at 4°C. After washing in 0.05 M phosphate-buffered saline (PBS), the sections were incubated with biotinylated anti-rat IgG (Vector Laboratories, Inc., Burlingame, CA) for 1 h at room temperature and then incubated with ABC reagent (Vector Laboratories) for 1 h at room temperature. The sections were then washed in PBS, incubated with 0.02% diaminobenzidine and 0.003% hydrogen peroxide in 0.1 M Tris-HCl-buffered saline (pH 7.5) for 5 min, rinsed with PBS, mounted on gelatin-coated slides, air-dried, dehydrated, and coverslipped.

The histological pictures were taken using a bright-field, phase contrast Axio Scope A1 microscope (Carl Zeiss, Oberkochen, Germany), and Axiocam ICc3 camera (Carl Zeiss). The hippocampal cell death and the astrocyte activation were quantitated in terms of the optical density in the CA3 of the hippocampus using Image-pro plus 6.0 (Media Cybernetics, Silver Spring, MD, USA).

### 2.5. Two-Dimensional Gel Electrophoresis

Twenty-four hours after the KA injection, the mice in the saline, KA, and KA + HT8 groups (*n* = 3 from each group) were killed by CO_2_ gas; the brain was immediately and rapidly removed from the cranium, and the hippocampus was extracted, weighed and stored at −80°C until use.

The hippocampi were homogenized directly by motor-driven homogenizer (PowerGen125, Fisher Scientific, Pittsburgh, PA, USA) in sample lysis solution composed of 7 M urea, 2 M Thiourea containing 4% (w/v) 3-[(3-cholamidopropy) dimethyammonio]-1-propanesulfonate (CHAPS), 1% (w/v) dithiothreitol (DTT) and 2% (v/v) pharmalyte and 1 mM benzamidine. Proteins were extracted for one hour at room temperature with vortexing. After centrifugation at 15,000 ×g for one hour at 15°C, insoluble material was discarded and the soluble fraction was used for two-dimensional gel electrophoresis. Protein concentration was assayed by the Bradford method [8]. 

IPG dry strips (4–10 NL IPG, 24 cm, Genomine, Republic of Korea) were equilibrated for 12–16 hours with 7 M urea, 2 M thiourea containing 2% CHAPS, and 1% DTT, 1% pharmalyte, and respectively, loaded with 200 *μ*g of sample. Isoelectric focusing (IEF) was performed at 20°C using a Multiphor II electrophoresis unit and EPS 3500 XL power supply (Amersham Biosciences, Uppsala, Sweden) according to the manufacturer's instructions. For IEF, the voltage was linearly increased from 150 to 3,500 V during 3 hours for sample entry followed by constant 3,500 V, with focusing complete after 96 kVh. Prior to the second dimension, strips were incubated for 10 minutes in equilibration buffer (50 mM Tris-Cl, pH6.8 containing 6 M urea, 2% SDS, and 30% glycerol), first with 1% DTT and second with 2.5% iodoacetamide. Equilibrated strips were inserted onto SDS-PAGE gels (20 × 24 cm, 10–16%). SDS-PAGE was performed using the Hoefer DALT 2D system (Amersham Biosciences) according to the manufacturer's instructions. 2D gels were run at 20°C for 1,700 Vh, and the 2D gels were then stained with Coomassie G250 as described by Anderson et al. [9].

Quantitative analysis of digitized images was carried out using the PDQuest (version 7.0, Bio-Rad, Hercules, CA, USA) software according to the protocols provided by the manufacturer. The quantity of each spot was normalized to total valid spot intensity. Protein spots that deviated over 1.4-fold in expression level compared with control or normal sample were selected for the significant expression variation.

### 2.6. Peptide Mass Fingerprinting

For protein identification by peptide mass fingerprinting (PMF), protein spots were excised, digested with trypsin (Promega, Madison, WI, USA), mixed with *α*-cyano-4-hydroxycinnamic acid in 50% acetonitrile/0.1% trifluoroacetic acid, and subjected to MALDI-TOF analysis (Microflex LRF 20, Bruker Daltonics, Billerica, MA, USA) as described by Fernandez et al. [10]. Spectra were collected from 300 shots per spectrum over *m*/*z* range 600–3000 and calibrated by two-point internal calibration using trypsin autodigestion peaks (*m*/*z* 842.5099, 2211.1046). The peak list was generated using Flex Analysis 3.0. The threshold used for peak picking was as follows: 500 for minimum resolution of monoisotopic mass, 5 for S/N. The search program MASCOT, developed by Matrix science (http://www.matrixscience.com/), was used for protein identification by PMF. The following parameters were used for the database search: trypsin as the cleaving enzyme, a maximum of one missed cleavage, iodoacetamide (Cys) as a complete modification, oxidation (Met) as a partial modification, monoisotopic masses, and a mass tolerance of ±0.1 Da. The PMF acceptance criterion was probability scoring.

### 2.7. Western Blotting

The anti-PURA primary antibody was purchased from Cell Signaling Technology (Beverly, MA, USA) and the anti-PP5 antibody was from Abcam (Cambridge, UK). For the Western blot, samples (50 mg protein) were loaded on 10% SDS-PAGE. After separation, the proteins were transferred to NC membrane. The membrane was shaken for 1 h at RT in TBS that contained 0.1% Tween-20, 5% skim milk, and 0.2% BSA. The membrane was incubated for 1 h at RT with primary antibodies (anti-PURA or anti-PP5, 1 :  1000) in TBS that contained 0.1% Tween-20. The primary antibodies were detected with an HRP-conjugated secondary antibody (anti-rabbit, 1 : 1000) and then visualized with ECL (Pierce, Rockford, IL, USA). These blots were then reprobed with an anti-*β*-actin antibody (1 : 1000; Santa Cruz Biotechnology, Santa Cruz, CA, USA). The band intensities of the detected proteins were measured by densitometry.

### 2.8. Statistical Analysis

All data are expressed as the mean ± S.E.M. and analyzed by one-way ANOVA with the Neuman-Keuls posthoc test. All statistical testing was performed using Prism 5 for Windows (GraphPad Software Inc., La Jolla, CA, USA). Statistical significance was set at *P* < 0.05.

## 3. Results

### 3.1. Effect of Acupuncture on KA-Induced Hippocampal Cell Death

**Figure 1:**
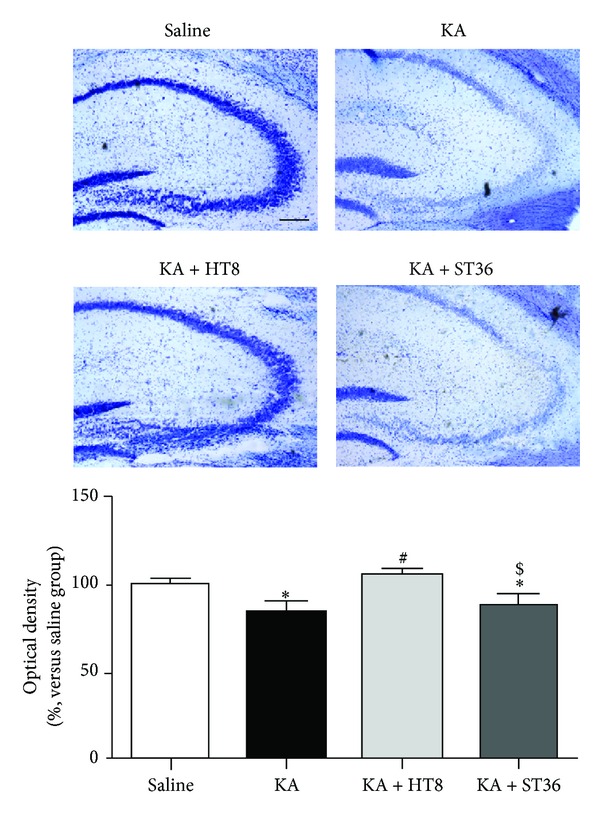
Neuroprotective effect of acupuncture stimulation at acupoint HT8 in hippocampal CA3 region after kainic acid (KA) administration. KA destroys neurons in the hippocampus, whereas acupuncture stimulation at HT8 prevents this destruction. Saline: the saline-injected control group; KA, the KA-injected group; KA + HT8: the acupuncture at HT8 with KA injection group: and KA + ST36, the acupuncture at ST36 with KA injection group. Scale bar represents 200 *μ*m. Data are expressed as the mean ±S EM. *P < 0.05 versus the saline group. ^#^ *P* < 0.05 versus the KA group. ^$^ *P* < 0.05 versus the KA + HT8 group.

We assessed the KA-induced cell death in the CA3 using cresyl violet staining. As a result, the optical densities of the CA3 region in KA (84.40 ± 5.34%) and KA + ST36 (89.02 ± 5.18%) groups were significantly reduced (*P* < 0.05 versus saline group), whereas the density in the KA + HT8 group was significantly elevated (105.76 ± 3.25%) compared to that in the KA group (*P* < 0.05, Figure 1).

### 3.2. Effect of Acupuncture on GFAP Expression in the Hippocampus

**Figure 2:**
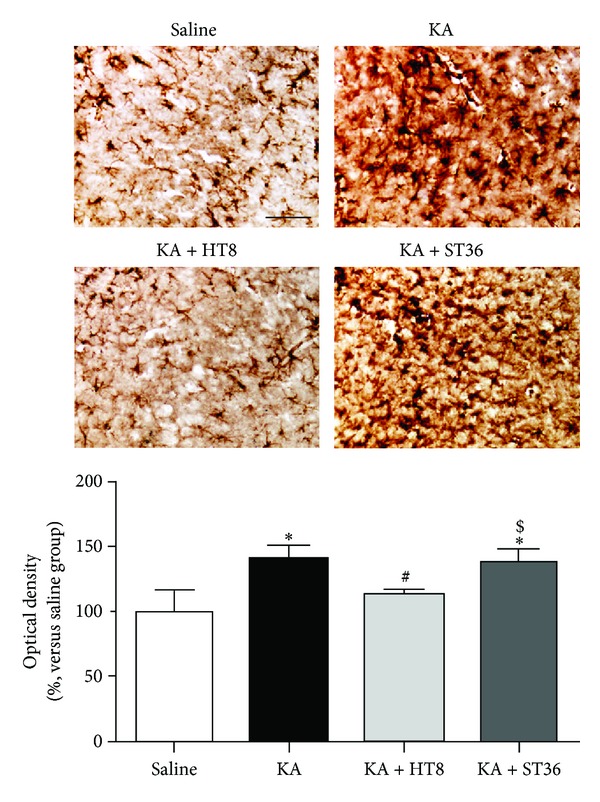
The activation of astrocytes in the hippocampus after kainic acid (KA) administration. KA upregulated GFAP activation in the hippocampus, whereas acupuncture stimulation at HT8 prevented this activation. Saline: the saline-injected control group; KA: the KA-injected group; KA + HT8, the acupuncture at HT8 with KA injection group; and KA + ST36: the acupuncture at ST36 with KA injection group. Scale bar represents 50 *μ*m. Data are expressed as the mean ± SEM. *P < 0.05 versus the saline group. ^#^ *P* < 0.05 versus the KA group. ^$^ *P* < 0.05 versus the KA + HT8 group.

The optical densities in the KA (140.45 ± 8.70%) and KA + ST36 (136.45 ± 9.80%) groups were significantly higher than those in the saline group (100.00 ± 14.54%, *P* < 0.05). However, the density was significantly lower in the KA + HT8 group (110.79 ± 5.93%) compared with that in the KA and KA + ST36 groups (*P* < 0.05 versus each group, Figure 2).

### 3.3. Proteins Differentially Expressed in the Hippocampus

**Figure 3:**
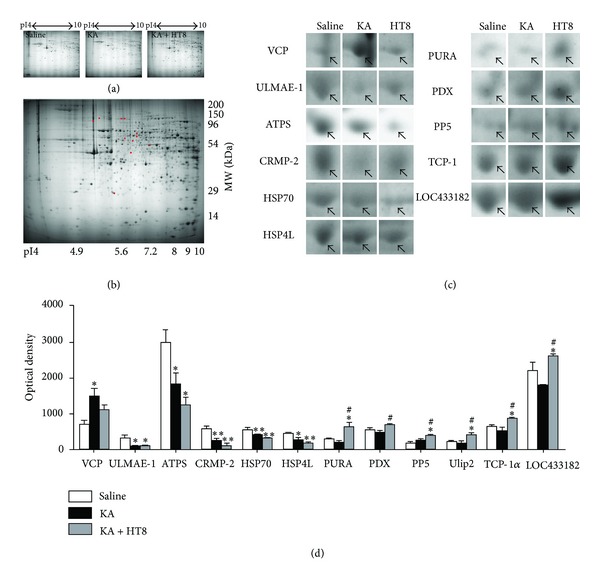
Protein profiles with differential expression. (a) Hippocampal tissue protein profiles obtained over different pI ranges. (b) Representation of identified differential protein spots (red) in 2-DE gel templates among the saline, KA and KA + HT8 groups. (c) Differential expression profiles of 11 proteins. Arrowheads on cropped images of 2-DE gels represent protein spots that showed different changes among the saline, KA and KA + HT8 groups. (d) Quantitative analyses of downregulated or upregulated proteins by kainic acid (KA) and acupuncture stimulation at HT8. The spot intensities were derived from Coomassie-stained 2-D gels. VCP: valosin containing protein; ULMAE-1, ubiquitin-like modifier-activating enzyme 1 isoform 1; ATPS: ATP synthase subunit d; HSP70: heat shock 70 kDa protein 4L; HSP4L: heat shock protein 4 like isoform CRA_c; CRMP-2: dihydropyrimidinase-related protein 2; PURA: transcriptional activator protein Pur-alpha; PDX: pyruvate dehydrogenase protein X component; PP5: serine/threonine-protein phosphatase 5; TCP-1*α*: T-complex protein 1 subunit alpha; LOC433182: uncharacterized protein LOC433182. Data are expressed as the mean ± SEM. *P < 0.05 and **P < 0.01 versus the saline group. ^#^ *P* < 0.05 versus the KA group.

To obtain the protein profiles of each group, 2-DE was performed with the protein extracts from the hippocampus. About 600 polypeptide spots could be revealed in the pH 3–10 interval with Coomassie G250 staining (Figures 3(a) and 3(b)). After matching the replicated maps, differential changes in intensities among the mice in the saline, KA and KA + HT8 groups were limited to 11 proteins: valosin containing protein (VCP), ubiquitin-like modifier-activating enzyme 1 isoform 1 (ULMAE-1), ATP synthase subunit d (ATPS), heat shock 70 kDa protein 4L (HSP70), heat shock protein 4 like, isoform CRA_c (HSP4L), dihydropyrimidinase-related protein 2 (CRMP-2), transcriptional activator protein pur-alpha (PURA), pyruvate dehydrogenase protein X component (PDX), serine/threonine-protein phosphatase 5 (PP5), T-complex protein 1 subunit alpha (TCP-1*α*), and uncharacterized protein LOC433182 (LOC433182). Compared to the expression of VCP in the saline group, that in the KA group was significantly increased (*P* < 0.05). That in the KA + HT8 group was also increased, but not significantly. The expressions of ULMAE-1, ATPS, HSP70, and HSP4L in the KA and KA + HT8 groups were significantly decreased compared to that in the saline group. CRMP-2 was actually decreased by KA injection (*P* < 0.01), but acupuncture stimulation at HT8 significantly restored it (*P* < 0.05 versus KA group). The levels of PURA, PDX, PP5, TCP-1*α*, and LOC433182 were unchanged by KA injection, but acupuncture stimulation at HT8 significantly increased them (Table 1, Figures 3(c) and 3(d)).

### 3.4. Confirmation of Altered Proteins by Western Blot Analysis

**Figure 4:**
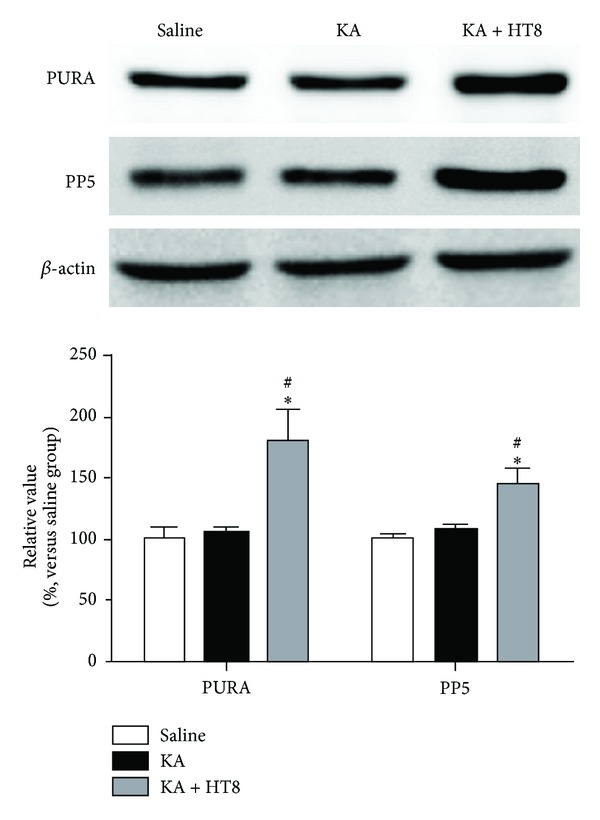
Validation of proteomic results using western blot analyses of PURA and PP5 proteins in the hippocampus of mice. The same trends detected in the 2-DE analyses were confirmed for these two proteins. PURA: transcriptional activator protein Pur-alpha. PP5: serine/threonine-protein phosphatase 5. Data are expressed as the mean ± SEM. *P < 0.05 versus the saline group. ^#^ *P* < 0.05 versus the KA group.

To verify the reliability of the proteomics analysis, PURA and PP5 were selected as representative proteins and subjected to western blotting. The results of triplicate western blots for the proteins using protein extracts from the hippocampus of mice in the saline, KA and KA + HT8 groups were consistent with those of the 2-DE (Figure 4).

## 4. Discussion

Our results demonstrate that acupuncture stimulation at HT8 protects against KA-induced neuronal death and astrocyte activation in the hippocampus. The protein expression profiles in the hippocampus of the saline, KA, KA + HT8 groups were compared using 2-DE, MALDI-TOF analysis, and peptide fingerprinting MS, and eleven proteins were differentially expressed.

During excitotoxin-induced neurodegeneration, microglia and astrocytes are activated, which contributes to hippocampal neuronal death through induction of inflammatory mediators [11]. KA is an excitotoxin, and the administration of KA causes epileptic seizures and activates microglia and astrocytes in the hippocampus. Therefore the deactivation of microglia and astrocytes plays an important role in reducing KA-induced neuronal cell death [12]. In this study, we confirmed that KA induces neuronal death and astrocyte activation in the hippocampus and that acupuncture stimulation at HT8 can suppress them. This result indicates that acupuncture stimulation at HT8 suppresses KA-induced neuronal cell death in the CA3 of the hippocampus, and the deactivation of astrocytes can account for the neuroprotective effect of the acupuncture stimulation in this study. 

VCP is an ubiquitin-dependent ATPase involved in protein lysis through the ubiquitin proteasome system associated with vesicle transport and fusion [13], 26S proteasome function, and peroxisome assembly [14]. It plays an important role in the ubiquitin-mediated protein degradation pathways in neurodegenerative disorders [15], and KA administration increases the level of VCP, which causes endoplasmic reticulum (ER) stress [16]. In this study, the administration of KA significantly increased the VCP expression; this increase was suppressed by acupuncture stimulation at HT8. Thus, acupuncture stimulation at HT8 may reduce KA-induced ER stress by suppressing VCP expression.

CRMP-2, which is in the collapsin response mediator protein family, induces axon guidance and growth and is the most abundant CRMP family member within the brain [17]. Changes in the level of CRMP-2 protein have been related to neurodegenerative disorders [18], and upregulation of CRMP-2 contributes to neuroprotective actions in reactive oxygen species (ROS) stress [19]. KA increases ROS production [20], therefore the upregulation of CRMP-2 may contribute to the suppression of neuronal death from KA toxicity. We found that acupuncture stimulation at HT8 restored the CRMP-2 level that was decreased by KA administration, which suggests that the restoration of the CRMP-2 level by the acupuncture stimulation contributed to the neuroprotective effect against KA toxicity.

PURA binds sequence-specific single-stranded deoxyribonucleic acid or ribonucleic acid and affects initiation of DNA replication and gene transcription, especially in the hippocampal CA3 region [21]. The decrease of PURA reduces postsynaptic density protein 95 [22], which contributes to neuronal cell loss after status epilepticus [23] and KA administration [24]. PP5, a serine/threonine phosphatase, is widely expressed in brain including hippocampus [25] and suppresses apoptosis by regulating c-Jun N-terminal kinase phosphorylation [26] and apoptosis signal-regulating kinase 1 activity [27]. T-complex protein 1 (TCP-1), a chaperonin family member, that folds protein properly [28], supports the maintenance of the native forms of cytoskeletal proteins [29], and the overexpression of all TCP-1 subunits suppressed Neuro2a cell death induced by the cytotoxicity of polyglutamine-expansion proteins [30]. Taken together, the increases of PURA, PP5, and TCP-1 have the potential to protect hippocampal neurons against KA toxicity. In this study, acupuncture stimulation at HT8 upregulated the levels of PURA, PP5, and TCP-1*α*, which may contribute to the suppression of hippocampal neuronal death induced by KA.

In conclusion, this study demonstrates that acupuncture stimulation at HT8 protects against KA-induced neuronal damage in the hippocampus, and that the acupuncture-mediated neuroprotection may be due, in part, to the normalization of the altered expression of VCP and CRMP-2, which are implicated in cell death mechanisms following KA administration. Furthermore, we propose that the PURA, PP5 and TCP-1*α* upregulation by acupuncture stimulation at HT8 may be a significant event in the neuroprotective effect of acupuncture.

## Figures and Tables

**Table 1:** Differentially expressed protein profiles of the protein spots after acupuncture stimulation at HT8.

Name	Theoretical		Gel		MASCOT score	Peptides matched	Sequence coverage (%)	Average ratio^a^		
	Mr (kD)	pI	Mr (kD)	pI				KA/Saline	KA + HT8/ KA	KA + HT8/ Saline
Valosin containing protein, isoform CRA_a	90.868	5.14	89.7	5.13	276	34	47	2.103*	−1.324	1.588
Ubiquitin-like modifier-activating enzyme 1 isoform 1	118.931	5.43	103.11	5.24	160	24	30	−3.576*	1.259	−2.841*
ATP synthase subunit d, mitochondrial	18.795	5.52	28.02	5.50	133	15	67	−1.631*	−1.471	−2.400*
Heat shock 70 kDa protein 4L	95.178	5.54	101.62	5.69	257	29	39	−1.321**	−1.245	−1.645**
Heat shock protein 4 like, isoform CRA_c	101.159	5.73	101.34	5.84	280	30	35	−1.718*	−1.480	−2.543**
Dihydropyrimidinase-related protein 2	62.638	5.95	56.96	5.87	210	26	53	−1.897**	1.417*	−1.339
Transcriptional activator protein Pur-alpha	34.976	6.07	46.54	6.20	110	11	38	−1.329	2.949*	2.220*
Pyruvate dehydrogenase protein X component, mitochondria	54.250	7.63	53.67	6.29	86	12	23	−1.189	1.523*	1.281
Serine/threonine-protein phosphatase 5	57.437	5.83	60.31	6.50	79	14	27	−1.293	1.688*	2.182*
T-complex protein 1 subunit alpha	60.867	5.82	64.14	6.53	177	21	44	−1.232	1.683*	1.366*
Uncharacterized protein LOC433182	47.453	6.37	50.76	7.20	221	24	55	−1.120	1.458*	1.302*

Saline: saline-injected group; KA: KA-injected group; KA + HT8: KA-injected group with acupuncture stimulation at HT8 acupoint. ^a^Average ratio calculated considering 3 replicate gels. A minus means that the protein expression was decreased. *P < 0.05 and **P < 0.01 between the groups.
